# 2-Diazo-1-(1,1-dioxothio­morpholin-4-yl)ethanone

**DOI:** 10.1107/S1600536811023774

**Published:** 2011-06-30

**Authors:** Åsmund Kaupang, Carl Henrik Görbitz, Tore Hansen

**Affiliations:** a Department of Chemistry, University of Oslo, PO Box 1033 Blindern, N-0315 Oslo, Norway

## Abstract

In the mol­ecule of the title compound, C_6_H_9_N_3_O_3_S, at 105 K, the six-membered ring is predominantly found in the chair conformation, with 1.89 (14)% in the boat conformation. In the crystal structure, there are five inter­molecular C—H⋯O=C and C—H⋯O=S contacts less than 2.6 Å, as well as a weak C—H⋯N=N inter­action to the diazo group.

## Related literature

For related structures found in the Cambridge Structural Database (Version 5.32 of November 2010; Allen, 2002 ▶), see: Fenlon *et al.* (2007 ▶); Haynes *et al.* (2006 ▶); Wang *et al.* (2006 ▶); Miller *et al.* (1991 ▶); Foces-Foces *et al.* (1988 ▶); Ganguly *et al.* (1980 ▶); Herdklotz & Sass (1969 ▶). For details of the synthesis, see: Kaupang (2010 ▶); Toma *et al.* (2007 ▶) and for the synthesis of related diazo­acetamides, see: Kaupang *et al.* (2010 ▶); Kaupang (2010 ▶); Ouihia *et al.* (1993 ▶). For quantum chemical calculations involving the acetamide analogue of the title compound, see: Fraenkel *et al.* (1992 ▶). For the Chemical Abstracts Service, see: American Chemical Society (2008 ▶). For hydrogen-bond graph-set notation, see: Etter *et al.* (1990 ▶).
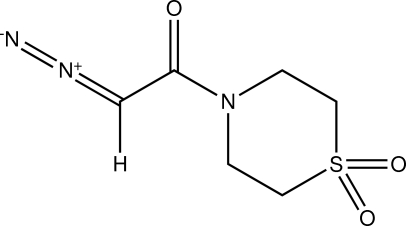


## Experimental

### 

#### Crystal data

C_6_H_9_N_3_O_3_S
*M* *_r_* = 203.22
Monoclinic, 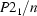

*a* = 5.2729 (3) Å
*b* = 20.1349 (11) Å
*c* = 7.8683 (4) Å
β = 95.171 (2)°
*V* = 831.97 (8) Å^3^
*Z* = 4
Mo *K*α radiation
μ = 0.37 mm^−1^
*T* = 105 K
0.80 × 0.30 × 0.20 mm

#### Data collection

Bruker APEXII CCD diffractometer
Absorption correction: multi-scan (*SADABS*; Bruker, 2007 ▶) *T*_min_ = 0.849, *T*_max_ = 0.929
14742 measured reflections
3951 independent reflections
3029 reflections with *I* > 2σ(*I*)
*R*_int_ = 0.031

#### Refinement

*R*[*F* ^2^ > 2σ(*F* ^2^)] = 0.056
*wR*(*F* ^2^) = 0.165
*S* = 1.11
3951 reflections
138 parameters
13 restraints
H atoms treated by a mixture of independent and constrained refinement
Δρ_max_ = 1.24 e Å^−3^
Δρ_min_ = −0.71 e Å^−3^

### 

Data collection: *APEX2* (Bruker, 2007 ▶); cell refinement: *SAINT-Plus* (Bruker, 2007 ▶); data reduction: *SAINT-Plus*; program(s) used to solve structure: *SHELXTL* (Sheldrick, 2008 ▶); program(s) used to refine structure: *SHELXTL*; molecular graphics: *SHELXTL*; software used to prepare material for publication: *SHELXTL*.

## Supplementary Material

Crystal structure: contains datablock(s) I, global. DOI: 10.1107/S1600536811023774/si2361sup1.cif

Structure factors: contains datablock(s) I. DOI: 10.1107/S1600536811023774/si2361Isup2.hkl

Supplementary material file. DOI: 10.1107/S1600536811023774/si2361Isup3.cml

Additional supplementary materials:  crystallographic information; 3D view; checkCIF report

## Figures and Tables

**Table 1:** Hydrogen-bond geometry (Å, °)

*D*—H⋯*A*	*D*—H	H⋯*A*	*D*⋯*A*	*D*—H⋯*A*
C2—H2⋯O2^i^	0.94 (2)	2.42 (2)	3.326 (2)	163 (2)
C3—H32⋯O1^ii^	0.99	2.47	3.372 (2)	152
C5—H52⋯O1^iii^	0.99	2.36	3.022 (2)	123
C5—H51⋯O3^iv^	0.99	2.56	3.445 (2)	149
C5—H52⋯N3^v^	0.99	2.59	3.417 (3)	141
C6—H61⋯O2^vi^	0.99	2.45	3.168 (2)	129

Symmetry codes: (i) 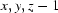; (ii) 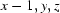; (iii) 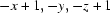; (iv) 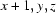; (v) 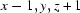; (vi) 
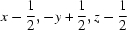
.

**Fig. 1.**
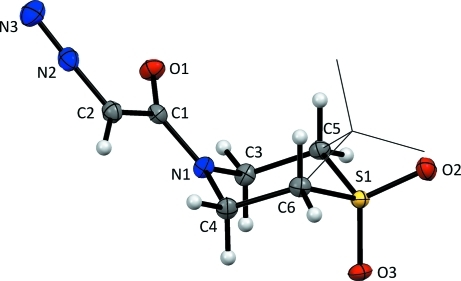
The asymmetric unit of (I) with atomic numbering indicated. Displacement ellipsoids are shown at the 50% probability level with H atoms as spheres of arbitrary size. The wireframe structure represents the minor boat conformation [occupancy 0.189 (14)].

**Fig. 2.**
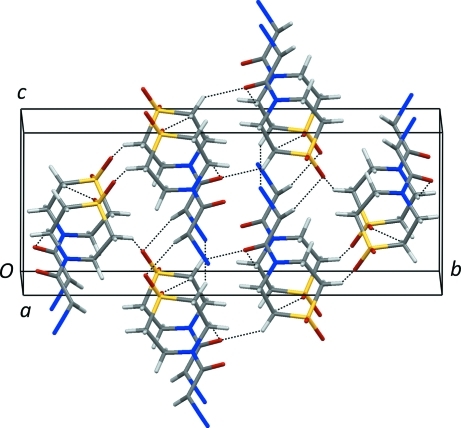
The unit cell and crystal packing of (I) viewed approximately along the a axis. A three-dimensional network of weak intermolecular C=O···H, S=O···H and N=N···H contacts with O/N···H < 2.6 Å are shown as dotted lines.

## supplementary crystallographic information

### Comment

2-Diazo-1-(1,1-dioxido-4-thiomorpholinyl)ethanone (I) was prepared as part of a series of diazoacetamides used in the intramolecular C—H insertion reactions taking place upon thermolysis of their corresponding α-bromodiazoacetamides (Kaupang, 2010). The title compound was synthesized from 2-bromo-1-(1,1-dioxido-4-thiomorpholinyl)ethanone following a procedure reported by Toma *et al.* (2007), modified to employ 1,1,3,3-tetramethylguanidine as the base instead of 1,8-diazabicyclo[5.4.0]undec-7-ene. No previous reports of this compound were found in the Chemical Abstracts Service (CAS, American Chemical Society, 2008).

In the crystal (I) occurs in the expected chair conformation, with a very minor fraction [occupancy 0.0189 (14)] in a boat conformation as shown in Fig. 1. The unit cell and crystal packing are depicted in Fig. 2, while a list of six C—H···O/N interactions with H···O distance shorter than 2.60 Å are given in Table 1. The two shortest interactions, with C2—H2 and C5—H52 donors, form *C*(8) chains along the *a* axis and dimeric *R*_2_^2^(12) ring motifs, respectively (Etter *et al.*, 1990). H52 is also close to the diazo N3 atom and thus participates in a three-center interaction. The interactions of (I) are different from those observed in our previous, related structure of *tert*-butyl 4-(2-diazoacetyl)piperazine-1-carboxylate (Kaupang *et al.*, 2010) where the H of the diazoacetyl group was accepted by the O atom of the same group.

### Experimental

A solution of 4.0 mg of the title compound in 500 µ*L* of CH_2_Cl_2_ was placed in a 2.5 ml vial which was capped and a pinhole (0.5 mm) made in the cap to allow for slow evaporation in darkness at ambient temperature. A single cluster of pale yellow needles appeared after approximately 48 h.

### Refinement

H atoms were positioned with idealized geometry and with fixed C—H distances at 0.99 Å, except H atoms bonded to C2 and C4 for which coordinates were refined, as too short intramolecular H···H distances resulted from putting these H atoms in theoretical positions. Distance restraints were imposed on the C2—H2 and C4—H41/H42 bonds utilizing *SHELX DFIX* 0.95 0.02 and *DFIX* 0.99 0.02 commands, respectively. Heavy atoms were refined anisotropically, except S11, O12 and O13 associated with the minor boat conformation of the six-membered ring with an occupancy 0.0189 (14). The first two were assigned *U*_iso_-values equal to the *U*_eq_-values of the corresponding atoms of the major chair conformation, while O13 received the same set of anisotropic thermal parameters as O2, from which it is separated by 0.35 Å. Failure to include the 2% minor conformation in the refinement increases the *R*-factor from 0.056 to 0.058 for 138 *versus*. 127 refinement parameters.

Initial checkCIF/PLATON results indicated possible twinning; introduction of the suggested command TWIN -1 0 0 0 - 1 0 0.269 0 1 during refinement gave a very modest decrease in the *R*-factor, from 0.057 to 0.056, with BASF = 0.00248.

### Crystal data

**Table:**

C_6_H_9_N_3_O_3_S	*F*(000) = 424
*M**_r_* = 203.22	*D*_x_ = 1.622 Mg m^−^^3^
Monoclinic, *P*2_1_/*n*	Melting point = 438–449 K
Hall symbol: -P 2yn	Mo *K*α radiation, λ = 0.71073 Å
*a* = 5.2729 (3) Å	Cell parameters from 5309 reflections
*b* = 20.1349 (11) Å	θ = 2.8–36.6°
*c* = 7.8683 (4) Å	µ = 0.37 mm^−^^1^
β = 95.171 (2)°	*T* = 105 K
*V* = 831.97 (8) Å^3^	Needle, yellow
*Z* = 4	0.80 × 0.30 × 0.20 mm

### Data collection

**Table:**

Bruker APEXII CCD diffractometer	3951 independent reflections
Radiation source: fine-focus sealed tube	3029 reflections with *I* > 2σ(*I*)
graphite	*R*_int_ = 0.031
Detector resolution: 8.3 pixels mm^-1^	θ_max_ = 36.8°, θ_min_ = 2.0°
Sets of exposures each taken over 0.5° ω rotation scans	*h* = −8→8
Absorption correction: multi-scan (*SADABS*; Bruker, 2007)	*k* = −30→32
*T*_min_ = 0.849, *T*_max_ = 0.929	*l* = −11→13
14742 measured reflections	

### Refinement

**Table:**

Refinement on *F*^2^	Primary atom site location: structure-invariant direct methods
Least-squares matrix: full	Secondary atom site location: difference Fourier map
*R*[*F*^2^ > 2σ(*F*^2^)] = 0.056	Hydrogen site location: inferred from neighbouring sites
*wR*(*F*^2^) = 0.165	H atoms treated by a mixture of independent and constrained refinement
*S* = 1.11	*w* = 1/[σ^2^(*F*_o_^2^) + (0.0728*P*)^2^ + 1.0103*P*] where *P* = (*F*_o_^2^ + 2*F*_c_^2^)/3
3951 reflections	(Δ/σ)_max_ < 0.001
138 parameters	Δρ_max_ = 1.24 e Å^−^^3^
13 restraints	Δρ_min_ = −0.71 e Å^−^^3^

### Special details

**Table:**

Geometry. All e.s.d.'s (except the e.s.d. in the dihedral angle between two l.s. planes) are estimated using the full covariance matrix. The cell e.s.d.'s are taken into account individually in the estimation of e.s.d.'s in distances, angles and torsion angles; correlations between e.s.d.'s in cell parameters are only used when they are defined by crystal symmetry. An approximate (isotropic) treatment of cell e.s.d.'s is used for estimating e.s.d.'s involving l.s. planes.
Refinement. Refinement of *F*^2^ against ALL reflections. The weighted *R*-factor *wR* and goodness of fit *S* are based on *F*^2^, conventional *R*-factors *R* are based on *F*, with *F* set to zero for negative *F*^2^. The threshold expression of *F*^2^ > 2σ(*F*^2^) is used only for calculating *R*-factors(gt) *etc*. and is not relevant to the choice of reflections for refinement. *R*-factors based on *F*^2^ are statistically about twice as large as those based on *F*, and *R*- factors based on ALL data will be even larger.

### Fractional atomic coordinates and isotropic or equivalent isotropic displacement parameters (Å^2^)

**Table:**

	*x*	*y*	*z*	*U*_iso_*/*U*_eq_	Occ. (<1)
S1	0.12602 (8)	0.17713 (2)	0.58307 (5)	0.01306 (11)	0.9811 (14)
O3	−0.1485 (2)	0.17127 (7)	0.56520 (19)	0.0176 (3)	0.9811 (14)
O2	0.2367 (3)	0.21336 (7)	0.73056 (18)	0.0194 (3)	0.9811 (14)
C5	0.2645 (3)	0.09728 (9)	0.5781 (2)	0.0152 (3)	0.9811 (14)
H51	0.4524	0.1012	0.5913	0.018*	0.9811 (14)
H52	0.2115	0.0703	0.6739	0.018*	0.9811 (14)
C6	0.2280 (3)	0.21174 (9)	0.3937 (2)	0.0156 (3)	0.9811 (14)
H61	0.1529	0.2565	0.3747	0.019*	0.9811 (14)
H62	0.4157	0.2163	0.4051	0.019*	0.9811 (14)
S11	0.385 (3)	0.1792 (3)	0.5843 (11)	0.013*	0.0189 (14)
O12	0.6562 (19)	0.1779 (9)	0.569 (4)	0.018*	0.0189 (14)
O13	0.302 (7)	0.2139 (3)	0.7308 (6)	0.0194 (3)	0.0189 (14)
C15	0.2645 (3)	0.09728 (9)	0.5781 (2)	0.0152 (3)	0.0189 (14)
H151	0.3969	0.0687	0.6379	0.018*	0.0189 (14)
H152	0.1168	0.0968	0.6474	0.018*	0.0189 (14)
C16	0.2280 (3)	0.21174 (9)	0.3937 (2)	0.0156 (3)	0.0189 (14)
H161	0.0734	0.2348	0.4255	0.019*	0.0189 (14)
H162	0.3408	0.2463	0.3519	0.019*	0.0189 (14)
O1	0.5857 (3)	0.02988 (7)	0.23496 (18)	0.0199 (3)	
N1	0.2562 (3)	0.10078 (8)	0.26290 (19)	0.0153 (3)	
C3	0.1799 (3)	0.06335 (9)	0.4088 (2)	0.0158 (3)	
H31	0.2552	0.0183	0.4081	0.019*	
H32	−0.0077	0.0585	0.3982	0.019*	
C4	0.1452 (4)	0.16671 (9)	0.2410 (2)	0.0175 (3)	
H41	−0.042 (3)	0.1625 (15)	0.232 (4)	0.021*	
H42	0.197 (5)	0.1864 (13)	0.135 (3)	0.021*	
C1	0.4582 (3)	0.07843 (9)	0.1828 (2)	0.0146 (3)	
C2	0.5141 (4)	0.11299 (10)	0.0274 (2)	0.0173 (3)	
H2	0.410 (5)	0.1428 (12)	−0.039 (3)	0.021*	
N2	0.7103 (3)	0.08892 (9)	−0.0445 (2)	0.0200 (3)	
N3	0.8809 (4)	0.06859 (12)	−0.1020 (2)	0.0294 (4)	

### Atomic displacement parameters (Å^2^)

**Table:**

	*U*^11^	*U*^22^	*U*^33^	*U*^12^	*U*^13^	*U*^23^
S1	0.01136 (17)	0.01274 (19)	0.01499 (19)	0.00030 (13)	0.00065 (12)	−0.00052 (13)
O3	0.0102 (5)	0.0198 (6)	0.0230 (6)	0.0005 (4)	0.0021 (4)	0.0000 (5)
O2	0.0209 (6)	0.0185 (6)	0.0182 (6)	−0.0011 (5)	−0.0011 (5)	−0.0042 (5)
C5	0.0151 (7)	0.0143 (7)	0.0163 (7)	0.0017 (5)	0.0013 (5)	0.0016 (5)
C6	0.0160 (7)	0.0132 (7)	0.0175 (7)	0.0001 (5)	0.0010 (5)	0.0017 (5)
O13	0.0209 (6)	0.0185 (6)	0.0182 (6)	−0.0011 (5)	−0.0011 (5)	−0.0042 (5)
C15	0.0151 (7)	0.0143 (7)	0.0163 (7)	0.0017 (5)	0.0013 (5)	0.0016 (5)
C16	0.0160 (7)	0.0132 (7)	0.0175 (7)	0.0001 (5)	0.0010 (5)	0.0017 (5)
O1	0.0195 (6)	0.0180 (6)	0.0224 (6)	0.0039 (5)	0.0026 (5)	0.0040 (5)
N1	0.0182 (6)	0.0130 (6)	0.0151 (6)	0.0021 (5)	0.0032 (5)	0.0019 (5)
C3	0.0177 (7)	0.0132 (7)	0.0168 (7)	−0.0010 (5)	0.0036 (5)	0.0008 (5)
C4	0.0207 (8)	0.0146 (8)	0.0169 (7)	0.0038 (6)	0.0005 (6)	0.0013 (5)
C1	0.0151 (7)	0.0144 (7)	0.0140 (7)	−0.0013 (5)	−0.0003 (5)	−0.0004 (5)
C2	0.0186 (7)	0.0190 (8)	0.0143 (7)	−0.0008 (6)	0.0013 (5)	0.0007 (6)
N2	0.0199 (7)	0.0243 (8)	0.0160 (7)	−0.0043 (6)	0.0022 (5)	0.0005 (5)
N3	0.0240 (8)	0.0410 (11)	0.0245 (9)	0.0015 (8)	0.0087 (7)	0.0003 (7)

### Geometric parameters (Å, °)

**Table:**

S1—O3	1.4463 (13)	O1—C1	1.235 (2)
S1—O2	1.4482 (14)	N1—C1	1.362 (2)
S1—C5	1.7679 (18)	N1—C4	1.455 (2)
S1—C6	1.7718 (18)	N1—C3	1.460 (2)
C5—C3	1.528 (3)	C3—H31	0.9900
C5—H51	0.9900	C3—H32	0.9900
C5—H52	0.9900	C4—H41	0.984 (17)
C6—C4	1.537 (3)	C4—H42	0.983 (16)
C6—H61	0.9900	C1—C2	1.460 (2)
C6—H62	0.9900	C2—N2	1.316 (2)
S11—O12	1.446 (2)	C2—H2	0.940 (17)
S11—O13	1.448 (2)	N2—N3	1.120 (2)
			
O3—S1—O2	116.52 (9)	C4—N1—C3	115.30 (14)
O3—S1—C5	109.59 (8)	N1—C3—C5	112.05 (14)
O2—S1—C5	109.77 (9)	N1—C3—H31	109.2
O3—S1—C6	109.11 (9)	C5—C3—H31	109.2
O2—S1—C6	110.17 (9)	N1—C3—H32	109.2
C5—S1—C6	100.48 (8)	C5—C3—H32	109.2
C3—C5—S1	109.70 (12)	H31—C3—H32	107.9
C3—C5—H51	109.7	N1—C4—C6	111.28 (15)
S1—C5—H51	109.7	N1—C4—H41	108.6 (17)
C3—C5—H52	109.7	C6—C4—H41	108.7 (17)
S1—C5—H52	109.7	N1—C4—H42	109.1 (17)
H51—C5—H52	108.2	C6—C4—H42	110.1 (17)
C4—C6—S1	109.81 (12)	H41—C4—H42	109 (2)
C4—C6—H61	109.7	O1—C1—N1	122.16 (16)
S1—C6—H61	109.7	O1—C1—C2	120.72 (16)
C4—C6—H62	109.7	N1—C1—C2	117.09 (16)
S1—C6—H62	109.7	N2—C2—C1	114.24 (17)
H61—C6—H62	108.2	N2—C2—H2	116.1 (17)
O12—S11—O13	116.5 (3)	C1—C2—H2	128.1 (18)
C1—N1—C4	124.74 (15)	N3—N2—C2	178.3 (2)
C1—N1—C3	118.42 (15)		
			
O3—S1—C5—C3	59.85 (14)	C3—N1—C4—C6	62.7 (2)
O2—S1—C5—C3	−170.98 (12)	S1—C6—C4—N1	−60.49 (17)
C6—S1—C5—C3	−54.93 (13)	C4—N1—C1—O1	161.34 (18)
O3—S1—C6—C4	−59.84 (14)	C3—N1—C1—O1	−3.9 (3)
O2—S1—C6—C4	171.06 (12)	C4—N1—C1—C2	−20.7 (3)
C5—S1—C6—C4	55.31 (13)	C3—N1—C1—C2	174.07 (15)
C1—N1—C3—C5	103.74 (18)	O1—C1—C2—N2	−1.7 (3)
C4—N1—C3—C5	−62.8 (2)	N1—C1—C2—N2	−179.68 (16)
S1—C5—C3—N1	60.09 (17)	C1—C2—N2—N3	−53 (8)
C1—N1—C4—C6	−102.9 (2)		

### Hydrogen-bond geometry (Å, °)

**Table:**

*D*—H···*A*	*D*—H	H···*A*	*D*···*A*	*D*—H···*A*
C2—H2···O2^i^	0.94 (2)	2.42 (2)	3.326 (2)	163 (2)
C3—H32···O1^ii^	0.99	2.47	3.372 (2)	152
C5—H52···O1^iii^	0.99	2.36	3.022 (2)	123
C5—H51···O3^iv^	0.99	2.56	3.445 (2)	149
C5—H52···N3^v^	0.99	2.59	3.417 (3)	141
C6—H61···O2^vi^	0.99	2.45	3.168 (2)	129

Symmetry codes: (i) *x*, *y*, *z*−1; (ii) *x*−1, *y*, *z*; (iii) −*x*+1, −*y*, −*z*+1; (iv) *x*+1, *y*, *z*; (v) *x*−1, *y*, *z*+1; (vi) *x*−1/2, −*y*+1/2, *z*−1/2.

## References

[bb1] Allen, F. H. (2002). *Acta Cryst.* B**58**, 380–388. 10.1107/s0108768102003890 12037359

[bb2] American Chemical Society (2008). Chemical Abstracts Service, American Chemical Society, Columbus, OH, USA; accessed Apr 27, 2010.

[bb3] Bruker (2007). *APEX2*, *SAINT-Plus* and *SADABS* Bruker AXS Inc., Madison, Wisconsin, USA.

[bb4] Etter, M. C., MacDonald, J. C. & Bernstein, J. (1990). *Acta Cryst.* B**46**, 256–262. 10.1107/s0108768189012929 2344397

[bb5] Fenlon, T. W., Schwaebisch, D., Mayweg, A. V. W., Lee, V., Adlington, R. M. & Baldwin, J. E. (2007). *Synlett*, pp. 2679–2682.

[bb6] Foces-Foces, M. C., Cano, F. H., Claramunt, R. M., Fruchier, A. & Elguero, J. (1988). *Bull. Soc. Chim. Belg.* **97**, 1055–1066.

[bb7] Fraenkel, G., Kolp, C. J. & Chow, A. (1992). *J. Am. Chem. Soc.* **114**, 4307–4314.

[bb8] Ganguly, A. K., Liu, Y.-T., Sarre, O., Jaret, R. S., McPhail, A. T. & Onan, K. D. (1980). *Tetrahedron Lett.* **21**, 49, 4699–4702.

[bb9] Haynes, R. K., Fugmann, B., Stetter, J., Rieckmann, K., Heilmann, H.-D., Chan, H.-W., Cheung, M.-K., Lam, W.-L. & Wong, H.-N. (2006). *Angew. Chem. Int. Ed.* **45**, 2082–2088. 10.1002/anie.200503071 16444785

[bb10] Herdklotz, J. & Sass, R. L. (1969). *Acta Cryst.* B**25**, 1614–1620.

[bb11] Kaupang, Å. (2010). MSc thesis, University of Oslo, Norway. PDF available online at http://urn.nb.no/URN:NBN:no-26202 or through http://www.duo.uio.no/.

[bb12] Kaupang, Å., Görbitz, C. H. & Hansen, T. (2010). *Acta Cryst.* E**66**, o1299. 10.1107/S1600536810016211 PMC2979480 21579396

[bb13] Miller, R. D., Theis, W., Heilig, G. & Kirchmeyer, S. (1991). *J. Org. Chem.* **56**, 1453–1463.

[bb14] Ouihia, A., Rene, L., Guilhem, J., Pascard, C. & Badet, B. (1993). *J. Org. Chem.* **58**, 1641–1642.

[bb15] Sheldrick, G. M. (2008). *Acta Cryst.* A**64**, 112–122. 10.1107/S0108767307043930 18156677

[bb16] Toma, T., Shimokawa, J. & Fukuyama, T. (2007). *Org. Lett.* **9**, 3195–3197. 10.1021/ol701432k 17629296

[bb17] Wang, J., Zeng, T., Li, M.-L., Duan, E.-H. & Li, J.-S. (2006). *Acta Cryst.* E**62**, o2912–o2913.

